# Burn Wound Healing: Clinical Complications, Medical Care, Treatment, and Dressing Types: The Current State of Knowledge for Clinical Practice

**DOI:** 10.3390/ijerph19031338

**Published:** 2022-01-25

**Authors:** Agnieszka Markiewicz-Gospodarek, Małgorzata Kozioł, Maciej Tobiasz, Jacek Baj, Elżbieta Radzikowska-Büchner, Agata Przekora

**Affiliations:** 1 Department of Human Anatomy, Medical University of Lublin, 20-090 Lublin, Poland; agnieszka.markiewicz-gospodarek@umlub.pl; 2 Chair and Department of Medical Microbiology, Medical University of Lublin, 20-093 Lublin, Poland; malgorzata.koziol@umlub.pl; 3 Eastern Center for Burn Treatment and Reconstructive Surgery, 21-010 Łęczna, Poland; biker001@o2.pl; 4 Department of Plastic, Reconstructive and Maxillary Surgery, CSK MSWiA, 02-507 Warszawa, Poland; elzbieta.radzikowska@gmail.com; 5 Independent Unit of Tissue Engineering and Regenerative Medicine, Medical University of Lublin, 20-093 Lublin, Poland; agata.przekora-kusmierz@umlub.pl

**Keywords:** burn, care, wound healing, microbiology, treatment, wound dressing, burn injury

## Abstract

According to the World Health Organization (WHO), it is estimated that each year approximately 11 million people suffer from burn wounds, 180,000 of whom die because of such injuries. Regardless of the factors causing burns, these are complicated wounds that are difficult to heal and are associated with high mortality rates. Medical care of a burn patient requires a lot of commitment, experience, and multidirectional management, including surgical activities and widely understood pharmacological approaches. This paper aims to comprehensively review the current literature concerning burn wounds, including classification of burns, complications, medical care, and pharmacological treatment. We also overviewed the dressings (with an emphasis on the newest innovations in this field) that are currently used in medical practice to heal wounds.

## 1. Introduction

Globally, burns are the fourth most frequent type of injury, after traffic accidents, falls and physical violence [1]. Until the first half of the 20th century the treatment of burn patients was very limited, and patients very often died because of hypervolemic shock in the first days after injury [2]. The second half of the 20th century brought intensive development of regenerative medicine, burn therapy, and pharmacotherapy. Nevertheless, treating burn wounds remains a challenge. When admitting a patient to the ward, several factors should be considered, ranging from the cause of the injury, the extent of the lesions, and tissue penetration. Initial clinical assessment of the burn area is often difficult and may be associated with incorrect classification, especially for moderate-degree burns. With deeper lesions, patients often require surgical intervention with the excision of irreversibly changed tissue. Conservative treatment alone in the case of deep and extensive burn wounds is often insufficient due to the poor general condition of the patient [3]. Appropriate treatment of the injury translates into therapeutic success, but so far ideal dressings for burn wounds have not been developed and implemented on a large scale that would allow their complete self-healing without constant control and care. Currently, there are several hydrogels, hydrocolloid, and hydro fiber dressings available on the market [4,5]. Despite many preventive measures, various types of complications may arise during the therapeutic process. The moist wound environment itself promotes adhesion, the multiplication of microorganisms, and the development of infection, which can be both exogenous and endogenous. Additionally, the appearance of secondary infections negatively affects the general condition of the patient, prolongs hospitalization and convalescence, while significantly increasing the cost of treatment [6]. The aim of this review is to examine the literature concerning aspects of burn types, measurements of burn surface area, the most common complications during the healing process, new treatment methods, and their use in clinical practice.

## 2. Classification of Burns According to the Depth of the Wound

Superficial burns (I degree). Called erythema, these cover the epidermis, accompanied by redness, slight swelling, and pain that subsides after 48–72 h [7]. The damaged epidermis peels off after 5–10 days. There is no visible scar after this type of burn. Sunburn is the most common cause of this type of burn [8].

Partial-thickness superficial burns (II degree). In the course of this type of burn, the epidermis and the dermis are damaged [8]. Additionally, these burns can be divided into the so-called mosaic types.

IIA: The epidermis and the superficial layers of the dermis are degraded. The skin is painful. Accompanied by blisters due to the delamination of the epidermis from the basement membrane. The wound heals in 14–21 days [9].

IIB: The epidermis and layers of the dermis are degraded at different depths. The burn does not include parts of the islets of the epidermis from the hair follicles and sweat glands. The skin is red, moist, and painful [9]. Epidermal necrosis in the wound may cause disturbances in the epithelial process. Scars may remain. Healing takes 21–35 days. Within a few moments after burns, blisters filled with fluid form, which are prone to rupture [8]. These kinds of wounds require surgical excision and skin transplantation [7,9].

Full-thickness deep burns (III degree). The full thickness is degraded. Burned skin is dry and tough. The color of the skin is brown, bronze or red. A characteristic is the absence of pain. Surgical treatment, transplant, or reconstructive treatment is necessary [8].

**Figure 1:**
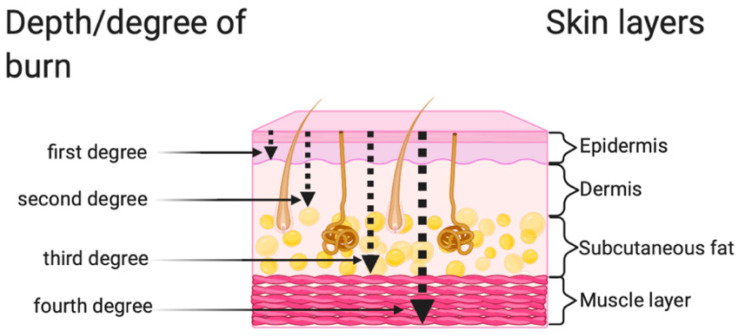
Classification of burn wound depth [7,8,9].

**Figure 2:**
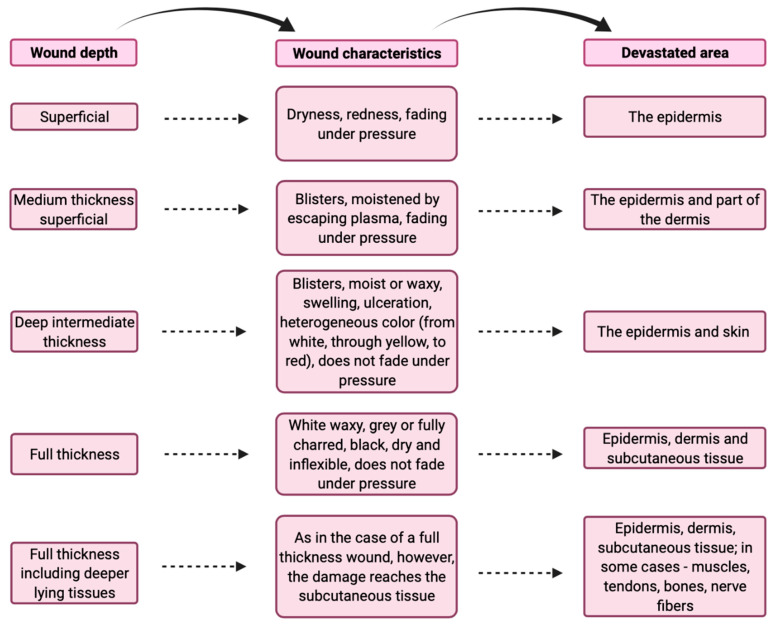
Characteristics of burns [10,11].

Full thickness including deeper lying tissues (IV degree). This is a mixed burn. It combines the features of second and third degree burns. These burns penetrate from the epidermis to the subcutaneous tissue layer, although in some patients muscle/bone involvement may occur, leading to local necrosis. These types of burns can be treated conservatively, and surgically [7,8]. All types of burns and their characteristics are presented in Figure 1 and Figure 2.

Determining the treatment procedure depends on the extent (area) and depth of the burn; therefore, the divisions of the American Burn Association should be considered.

Severe burns [12,13]:

2nd degree burns involving more than 25% of body surface area in adults.

2nd degree burns involving more than 20% of the body surface in children.

3rd degree burns affect more than 10% of the body surface.

Respiratory burns, electrical burns, burns complicated by the other major trauma.

Burns extensively affecting the hands, face, eyes, ears, feet, and perineum.

Medium burns:

2nd degree burns in adults covering 15–25% of the body surface.

2nd degree burns in children covering 10–20% of the body surface.

3rd degree burns affecting 2–10% of the body surface.

Light burns:

2nd degree burns in adults involving less than 15% of body surface.

2nd degree burns in children covering less than 10% of the body surface.

3rd degree burns covering less than 2% of the body surface.

**Figure 3:**
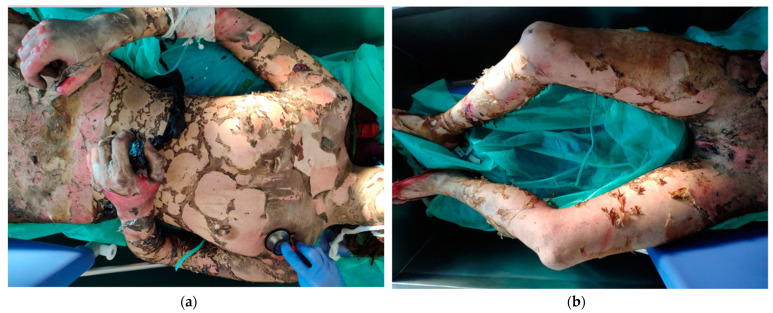
(**a**,**b**) 3rd/4th degree burns—suicide by arson.

**Figure 4:**
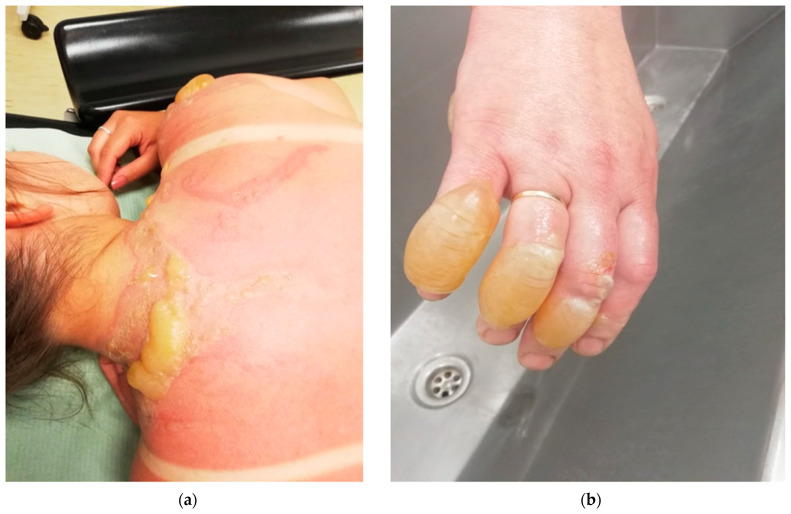
(**a**,**b**) 2nd degree burn—scalding with hot soup.

**Figure 5:**
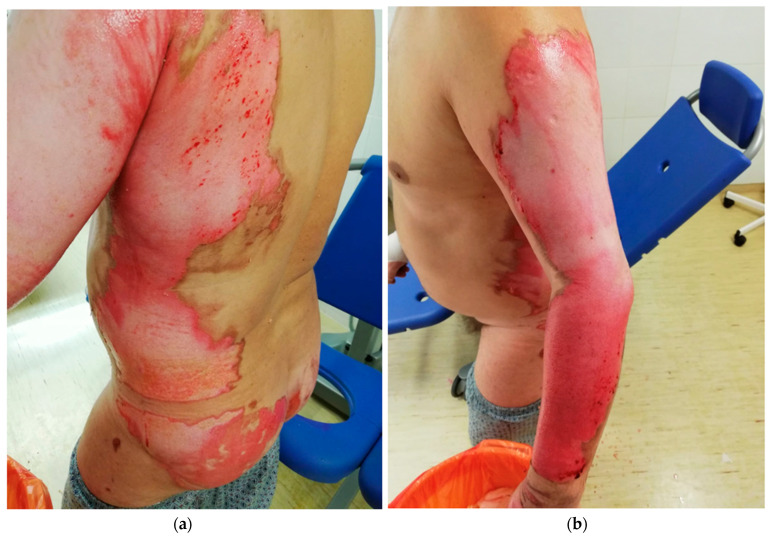
(**a**,**b**) 2nd degree burn.

**Figure 6:**
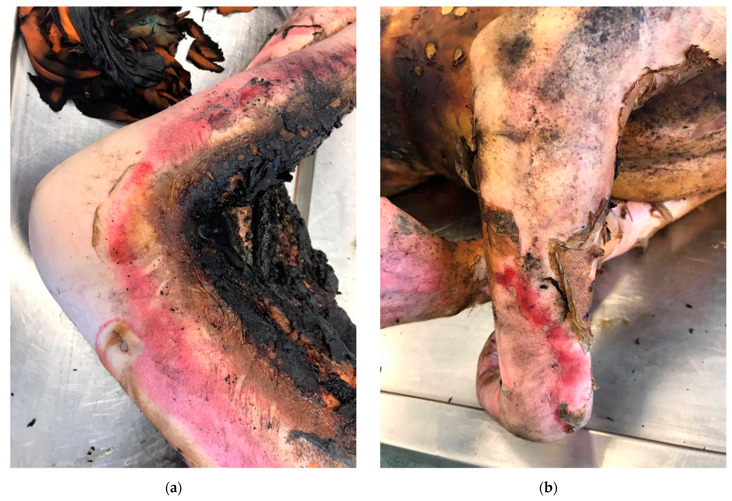
(**a**,**b**) 4th degree burn—carbon monoxide poisoning.

Individual types of burns are presented in the author’s photos (Figure 3a,b, Figure 4a,b, Figure 5a,b and Figure 6a,b) obtained from clinical practice.

Considering the type of burn, its depth, and the extent of the burned area there is a potential risk of hypertrophic scarring, i.e., scar tissue elevation [14]. Such a clinical condition is associated with a loss of skin aesthetic value, while histologically it consists of a hypercellular mass and disorganized connective tissue [15]. Ultrasounds have proven to be helpful in measuring this type of changes, i.e., in vivo skin thickness measurement [16]. It has been shown to be a sensitive, reliable, and repeatable examination method [17]. Compared to subjective clinical judgment it allows a quantitative measurement of the total thickness of scar tissue. This type of measurement allows for obtaining a lot of useful information such as the time to reach the maximum thickness after a burn, the thickness of the scar depending on the location and the efficacy of pressure garment therapy [18]. Additionally, there is the possibility of MoorLDI Doppler laser imaging in the context of burns [19]. It has been shown that the above method accurately predicts the healing time, enables an earlier decision on transplantation and ultimately results in a shorter hospital stay [14]. 

In addition to the above-mentioned hypertrophic scars, itching can occur through histamine production by mast cells present in the wound [20]. Histamine increases surface blood flow through the wound, contributing to the erythematous appearance of chronically itchy wound [21]. Acute burn pruritus occurs in the period from wound closure to the early remodeling phase of healing [22]. Post-burn pruritus is insufficiently studied but its severity is usually described as severe. Additionally, it is relatively difficult to treat with conventional methods such as topical corticosteroids [23]. In addition to itching there may be a variety of sensory ailments such as “pins and needles” stabbing or burning sensations and there may be behavioral problems that interfere with sleep and result in poor healing [22,24,25].

## 3. Complications in Burn Patients during the Healing Process

The process of burn wound healing is a complex long-lasting process that involves a few repair processes dependent on the immune system [26], and involves the reconstruction of broken tissue continuity resulting from a random event, e.g., a fire. For a burn wound to heal, three stages must occur successively: inflammation, granulation tissue formation (proliferation), and remodeling (which may result in scarring) [27]. The patient’s immune system plays the main role in wound healing. The immediate reaction after burns occurs involves a cascade of biological mediators of inflammation and growth factors: interleukins (IL-1, IL-2, IL-4, IL-8, IL-10), fibroblast growth factor (FGF), platelet-derived growth factor (PDGF), and different growth factors (EGF—epidermal growth factor, TGF—transforming growth factor, VEGF—vascular endothelial growth factor), interferon-gamma (INF-gamma), tumor necrosis factor (TNF alpha and beta), as well as many other cells of the immune system and elements of the extracellular matrix [4,28,29]. Thus, a critical role while healing is played by the stimulation or impairment of immune responses in the patients. An important aspect of the therapeutic process in a correct and effective manner is the coexistence of angiogenesis.

Before the injury (in this case, a burn) occurs, the vascular system remains at rest, and its blood vessels are properly perfused to provide the tissues with an adequate amount of oxygen and nutrients [30]. When tissue damage occurs, homeostasis is automatically broken, associated with fluid accumulation, inflammation and, ultimately, the development of hypoxia [31]. Consequently, the production of one of the most important angiogenic factors, VEGF, begins. This factor is responsible for the stimulation of capillaries to create new, immature loops and branches [32].

Patients with burns covering at least 15% of the total body surface area (TBSA) develop an acute phase of the immune response leading to extensive systemic inflammation, and multi-organ dysfunction [33]. The coexistence of the above-mentioned symptoms with bacteremia leads to sepsis and organ failure [34]. Burn patients, especially those with extensive and deep injuries, are often predisposed to septic complications, which are one of the biggest problems with modern medicine. Infectious complications increase the cost and the length of treatment, as well as mortality rates. Considering the general clinical condition of admitted patients, the fight against developing infections is a huge challenge that physicians face in everyday practice. This applies not only to changes directly occurring in the burn wound but also to systemic infections such as pneumonia, urinary tract infections, bacteremia, and sepsis. The infections can have exogenous and endogenous characters, and secondary effects due to spread of microorganisms. Therefore, it is important to identify the most common etiological agents of infection along with the mechanisms of drug resistance. External risk factors, such as supplying the burned area, as wells pathogen virulence factors and patient-related factors (modifiable/non-modifiable), e.g., age, obesity, diabetes, hypertension, play important roles in risk stratification. The key role in achieving rapid therapeutic success is to analyze risk factors, improve the method of monitoring the patient’s condition, search for new diagnostic laboratory markers and indicators of developing infection, and using modern preventive methods [35,36,37].

It seems that the skin and burn surface are the most susceptible places for infection complications, especially in the first week of hospitalization [1,38,39]. The data of *Junaidi* et al. confirmed the high capacity for contamination, microbial colonization, and then clinically visible development of infection at the site of injury, as culture of collected wound swabs were 94% positive [40]. Nevertheless, an analysis of research studies on infectious complications in burn patients showed that burn wound infection (BWI) was not the most common type of infection on the emerging scale. According to Ramirez-Blanco, Ramirez-Rivero, Diaz-Martinez, bacterial lesions in the wound affected only 4.2% of the analyzed group (patients with II/III-degree burn, >50 age, burns by fire), and skin graft infection appeared in 1.7% of cases [37].

Considering the spectrum of pathogens that colonize various types of wounds, and cause skin and soft tissue infections (SSTI) at surgical sites, Gram-positive cocci are common. Many cutaneous microorganisms that are components of skin microbiota, such as *Staphylococcus epidermidis*, can translocate, colonize the wound, and cause infection. The infection is then called endogenous and opportunistic. *Staphylococcus aureus* is another Gram-positive coccus that is common and a known pathogen. This bacterium contains numerous virulence factors, is particularly important in wound contamination, is a major agent of nosocomial infections, and can colonize the nasopharyngeal cavity. The presence of bacteria in the nasopharynx is an important risk factor for future infectious complications under certain circumstances. It is estimated that the carriage of *S. aureus* affects approximately 20–30% of people, and occurs in around 44% of medical personnel. While the carrier state in patients undergoing elective procedures is eradicated, it is different in emergency burn patients, where the probability of staphylococcal infection increases significantly [41,42]. Scientific reports indicate variable levels of BWI caused by *S. aureus*. The data presented by Bayram, Parlak, Aypak and Bayram show that *S. aureus* constituted only 11.2% of the obtained isolates [43]. Azimi, Moteballian, Namvar, Asghari and Lari, showed the pathogen was only third on the scale of all obtained wound isolates (~16%) [44], while another analysis by Junaidi, Mustafa, Arshadm, Al Farraj, Younas, and Ejaz put *Staphylococcus* in the second position (~29%) after Gram-negative rods cultured from a burn wound [40]. A slightly higher frequency was reported by Alebachew, Yismaw, Derabe, and Sisay in which patients with burns had an overall prevalence of *S. aureus* of 57.8% without a significant correlation with age and gender. Moreover, all strains were classified as multidrug-resistant (MDR) [45]. Multi-drug resistance was confirmed by the studies of Chen et al. in which the percentage of methicillin-resistant *S. aureus* (MRSA) strains was much higher in burn centers, especially if *Staphylococcus* was the cause of SSTIs, compared to other types of infections of this etiology [46]. MRSA strains are an urgent medical problem because the resistance mechanism precludes the use of almost all antibiotics from the β-lactam group (except the fifth generation of cephalosporin). Currently, it is recommended that vancomycin (glycopeptide), linezolid, daptomycin (lipopeptides), telavancin (lipoglycopeptide), or clindamycin are used in severe, complicated infections of soft tissues [42]. According to Bayram, Parlak, Aypak and Bayram analysis of staphylococci responsible for BWI (accounting for 19% of cases) shows that in the treatment of MRSA infection vancomycin, and linezolid were the most effective antibiotics [43]. As well, in patients with second and third-degree burns, mupirocin antibiotics may be used as an adjuvant for topical, and preventive treatment [44], which are relatively safe antibiotics effective against most aerobic Gram-positive bacteria, and particularly active against *S. aureus* and *S. epidermidis*, including methicillin-resistant strains. They are characterized by slow resistance development, though current widespread use is creating a risk of increasing bacterial resistance [41]. When selecting an antibiotic, it is worth considering the use of combination therapy depending on the exotoxins produced by *S. aureus* such as TSST-1 (Toxic Shock Syndrome Toxin), PVL (Panton Valentine Leukocidin), and ET (Epidermolytic Toxin/exfoliative). In this case, it is recommended to combine anti-staphylococcal penicillin with clindamycin (lincosamides) or linezolid (oxazolidinones) because they block the action of toxins [42].

When analyzing the changes taking place in the burnt surface, apart from the contamination of the wound with Gram-positive cocci in the initial phases, Gram-negative bacteria are more often involved in BWI in the wound healing process. They appear a bit later, but show high drug resistance [1,38,39]. Hard-to-heal wound and chronic wounds often have poly-microbial etiology. Growth in co-cultures is observed, and bacteria can create a biofilm structure. The moist wound environment enhances the rapid growth of bacteria, especially from the *Enterobacteriaceae* family that infect deeper skin layers. This significantly determines the available treatment options, considering the growing number of strains capable of producing carbapenems [38,39,45]. Bayram, Parlak, Aypak and Bayram showed that Gram-negative rods were dominant, such as *Acinetobacter baumannii* (23.6%), *Pseudomonas aeruginosa* (12%), and *Escherichia coli* (10%), and regardless of the degree of burn the etiology of infection was similar [43]. According to the suggestion of Azzopardi Azzopardi, Azzopardi, Camilleri, Villapalos, Boyce, Dziewulski, Dickson and Whitaker presented in a systematic review, a changeable spectrum of pathogens and BWI with Gram-negative etiology predominate, except in the early post-burn period. The bacteria do not differ as much between burn centers and include Gram-negative rods such as *Klebsiella pneumoniae*, *E. coli*, *Enterobacter* spp., *Proteus* spp., and *P. *aerugionsa** as the commonest BWI pathogens [47]. *Pseuodmonas* spp., especially *P. aerugniosa*, appears many times in the results of numerous studies. Like *A. baumanii*, this rod is a common pathogen occurring in intensive care units and burn treatment centers [48]. *Pseudomonas* spp. can survive in a hospital environment for a long time, creates a biofilm structure and, at the same time, quickly develops multi-drug resistance to commonly used antibiotics. It is an opportunistic pathogen; therefore, people who have lowered immunity, in combination with a damaged skin barrier resulting from burns, often become infected [48,49,50]. The presence of dead, denatured tissue and the moist wound environment makes the burn wound susceptible to *Pseudomonas* spp. infection. Contamination is facilitated by numerous virulence factors, including exotoxin A (ETA), exoenzyme S, elastase, sialidase activity, and expression of siderophores. It is believed that ETA is the major virulence factor produced by most of the isolated strains of *P. aeruginosa* [50,51].

A biofilm is a structure formed of a community of microbial cells. It is an important element disturbing the treatment process and constitutes a huge challenge for modern pharmacology. Developing on inanimate surfaces and dead tissues, it provides favorable conditions for the microorganism’s multiplication by a self-produced complex matrix of extracellular polymeric substances. The resulting bacterial mucus facilitates adherence to tissues and protects microorganisms from elements of the immune system, enables the activation of macrophages, and stimulates the production of inflammatory mediators such as IL-6 and TNF-α. The formation of a biofilm involves stages of attachment, multiplication, development and later dispersal. As a structure not susceptible to disinfectants and drugs. It allows pathogens to survive in the presence of commonly used antibiotics, even at concentrations much higher than standard doses [51,52]. Interesting research on biofilm creation in full-thickness scald burns has been presented by Brandenburg et al. By using a modified Walker-Mason’s rat scald burn model they checked how *P. aeruginosa* can readily form biofilms. They observed increased expression of bacterial pellicle biofilm matrix genes inside the burn scab, and up-regulation of alginate genes (alg8 and algE), and the iron-binding siderophore pyoverdine (pvdS), which are the molecules contributing to the biofilm matrix structure [51].

In addition to infectious complications in the burn wound, patients develop pneumonia, urinary tract infections (UTIs), and bloodstream infection (BSI). Considering that these develop later during hospitalization (>30 days after admission), infections can be classified as nosocomial [38]. According to Ramirez-Blanco, Ramirez-Rivero, Diaz-Martinez, 27.8% of patients had at least one episode of infection, including 7.7% of burn patients with UTI, 3.0% with BSI, and 3.0% had catheter-related BSI (CRBSI); only 3.5% developed pneumonia [37]. Pneumonia is related to prolonged mechanical ventilation, so patients present ventilator-associated pneumonia (VAP) and its frequency is variable in 20–40% of burn patients. Etiology is mostly Gram-negative [37,38].

Multiple infections are usually observed in patients with burn wounds. Patients with extensive damage to soft tissues may experience the spread of microorganisms and the development of secondary infection. The hematogenous spread of bacteria leads to bacteremia and sepsis progress [37]. Sepsis is a life-threatening condition caused by an inappropriate response to infection [53]. Currently, there is no universal diagnostic test for sepsis, and diagnosis is based on a combination of clinical and laboratory criteria [54]. Moreover, the diagnosis of sepsis in severely burned patients (>20% of the total body surface area of TBSA) is particularly difficult due to overlapping clinical signs of a hypermetabolic burn response with symptoms of sepsis [55]. To meet the demand for diagnostic criteria specific to burn patients, the American Burn Association (ABA) developed a burn-specific sepsis definition in 2007 [56]. Sepsis is a comorbid disease commonly observed in patients with severe burns and is the leading cause of death [57]. The latest reports show the incidence of sepsis in burn patients ranges from 8% to 42% and the related mortality from 28% to 65% [57]. According to the Centers for Disease Control and Prevention (2018) in the United States, each year around 11% of all diagnosed sepsis cases are a result of a burn-related skin infection, but sepsis can develop as a result of any other site infection [58]. The occurrence of sepsis in burn patients is caused by a depression of the immune response (cellular and humoral), a massive systemic inflammatory response (SIRS), and invasive types of bacteria [59]. Severe burns contribute to the appearance of sepsis as they promote the development of infection due to skin damage, necrosis, the use of catheters and other invasive methods, and exposure to unfavorable hospital flora [60]. Patient-related risk factors (modifiable/non-modifiable) such as age, comorbidities (obesity, diabetes, arterial hypertension), prior medical conditions, burn type, and response to proposed treatment, play an important role as well [61]. The scale of burn severity itself is still a prognostic factor for the development of complications in burn wards [56,61].

**Figure 7:**
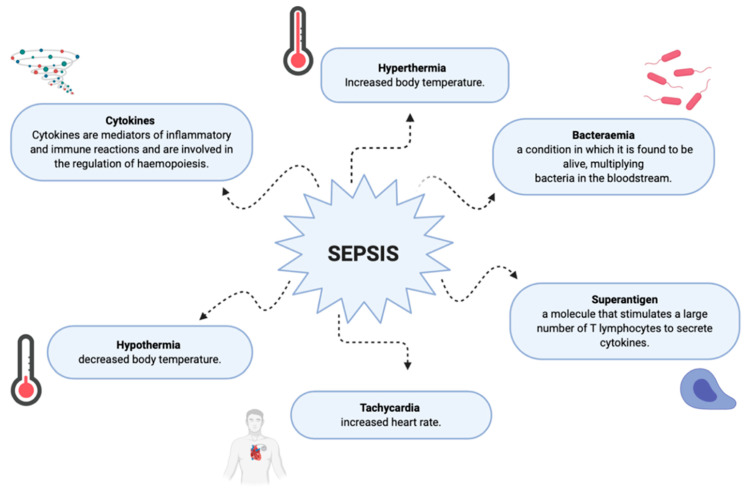
Potential symptoms of sepsis.

Musculoskeletal changes are another complication in burn patients that are worth mentioning because of their frequency. These include contractures, bone loss, heterotrophic ossification, scoliosis, kyphosis, and septic arthritis. They are a direct or indirect consequence of burn injury and have effects on bones, muscles, and tendons [62]. Deep skeletal muscle atrophy is the hallmark of massive burns that hamper recovery and require a longer period of rehabilitation and convalescence [63]. Figure 7 shows the potential sequelae, symptoms and effects of sepsis.

## 4. Oxidative Stress in Burned Patients

One of the important pathophysiological elements of burns is the development of oxidative stress. Perfusion of ischemic tissues after a thermal trauma results in an imbalance between reactive oxygen species (RTF), and the antioxidant defense system due to the excessive production of free radicals [64]. Therefore, oxidative stress is a state of imbalance between the action of RTF and the biological ability to quickly detoxify reactive intermediates and repair the damage that has occurred. Direct quantification of RTF is currently a major, but difficult, challenge due to the very short half-life of radicals and their extremely low concentrations in biological systems [65]. In the case of burns, RTF is an important factor that can increase vascular permeability and peroxidation of the plasma membrane lipids, and can cause local or systemic inflammation [66]. Lipid peroxidation is the leading mechanism of RTF cell damage due to the formation of aldehydes such as malondialdehyde (MDA) [65]. This compound is widely used as a biomarker in lipid peroxidation due to its reaction with thiobarbituric acid (TBA). In previous studies by Foldi et al. [67] it was also pointed out that burns may promote lipid peroxidation, i.e., an autocatalytic reaction initiating toxic metabolism and cell apoptosis [68]. Therefore, based on the research conducted by a team of scientists [68], to improve the effectiveness of treatment and prevent potential OFR injuries it is recommended that patients with burns use antioxidants, such as, e.g., thiopronine, as early as possible, due to its protective activity against oxidative tissue damage [69].

## 5. Care of a Patient with Burns

Care of burn patients requires a lot of experience and is based mainly on the depth of the injury. The person caring for the patient must consider not only the general condition of the patient but also act aseptically to prevent infection. As already mentioned, local or general immunosuppression, the extent of a burn wound, and the age of coexisting diseases, are important factors influencing the treatment process [70]. Prevention, effective control, and therapy of an infected burn wound requires close cooperation of medical personnel with the attending physician, and constant observation of the burn site is of key importance. The change in burn depth and burn conversion should be monitored, bearing in the mind the possibility of an unexpected discoloration of the necrosis which turns from pale or whitish to dark brown, black, or purple. According to the definition given by the Centers for Disease Control and Prevention (CDC) concerning skin and soft tissue infection with respect to burns, one of the following conditions must exist: a change in the appearance or nature of the burn wound after a burn such as a rapid separation scab, dark brown, black, or purple discoloration of the scab, edema of the wound edge, and invasion of the adjacent living tissue [70,71].

If the patient has undergone surgical intervention, with associated postsurgical pain, the discomfort should be minimized through the regular supply of drugs to maintain a constant concentration in a sample of blood. The time affecting the patient’s condition after severe burns is of key importance because severe burns result in significant damage to the intestinal mucosa and increased translocation of bacteria, which are closely related to impaired absorption of nutrients [72]. Preparations for the basic purpose of meeting caloric needs and providing building substances may have a greater stimulating effect on the immune system than before. In patients with burns, especially severe burns, catabolic processes are intensified and lead to a decrease in muscle mass, a negative nitrogen balance, and disturbance of the protein balance of the entire system [73]. The occurrence of the above-mentioned changes results in malnutrition of the patient due to their immobilization; therefore, close cooperation of the medical personnel with a clinical dietitian is recommended to minimize the negative effects of immobilization and select the appropriate nutrition of the patient. In addition, immobilization itself, which is primarily aimed at improving the condition of the patient after a severe injury, is associated with increased protein catabolism and increases the potential risk of infectious complications.

Patients who have developed severe burns have increased metabolic requirements (hypermetabolism), which is a characteristic feature of the stress reaction to burns [74]. In such cases, it is recommended to insert an enteric tube and start feeding as soon as possible, even during initial resuscitation [1]. *Mochizuki* et al. showed that guinea pigs that were continuously enteral fed starting 2 h after burns showed a significant decrease in metabolic rate 2 weeks after the burn, compared to those that started enteral feeling 3 days after the burn [75]. However, the administration of nutrients by the enteral route may have unfavorable effects, since such administration increases the oxygen demand of the gastrointestinal mucosa and intestinal villi [76]. If the increased oxygen demand is not met, musical necrosis may occur [77].

Another treatment, though rarely used, is to reduce catabolism and increase muscle mass through the provision of anabolic agents [74].

## 6. Pharmacological Treatment

Proper pharmacological treatment remains the most crucial aspect in the management of pain in burn patients. Of major importance in considering the pharmacological treatment of burn patients is individualized therapy depending on the patient’s current medical condition, pulmonary status, the severity of the injury, medications taken by the patients, and any concomitant disorders. The presence of severe burns is usually associated with altered pharmacokinetic and pharmacodynamic responses to a vast number of drugs; thus, the choice of a proper treatment usually constitutes a challenge for such patients [78]. The management of pain in burn patients just after an injury includes the administration of opioids, among which morphine usually constitutes the first line-treatment. Other opioids, including clonidine, ketamine, dexmedetomidine, and methadone have been found to be effective, especially in cases of patients with a lower tolerance to morphine [79,80]. In addition to analgesic properties, ketamine was observed to exhibit anti-inflammatory properties, which are desirable in the case of burn patients since they tend to present a significantly higher probability of post-traumatic sepsis [81]. Opioids combined with benzodiazepines are commonly used in those patients who exhibit high levels of procedural pain. A synergy with opioids was observed while administrating the alpha 2-agonists, dexmedetomidine and clozapine, while the latter was observed to be safe and effective in the case of pediatric burn patients [82,83]. Nevertheless, the administration of opioids should be minimized due to highly prevalent opioid-induced hyperalgesia as a side effect. While administrating paracetamol, dipyrone, or cyclooxygenase-2 inhibitors, the amounts of opioids needed, and potential side effects, can be reduced by about 20–30% [84]. The treatment of post-operative pain of minor burns includes the administration of acetaminophen or oral nonsteroidal anti-inflammatory drugs (NSAIDs); the latter usually combined with benzodiazepines [85]. Crucial is that burn patients usually develop anxiety or post-traumatic stress disorder (PTSD) induced by the trauma associated with the burn; thus, proper additional treatment of these conditions is highly important. Besides, anxiety might also exaggerate burn-related pain, and its reduction constitutes a crucial part of the multimodal management of burn patients. The combination of anxiolytic drugs and opioids was observed to provide beneficial effects in the case of burn patients. Antidepressants such as amitriptyline can also be beneficial in the reduction of neuropathic pain [86]. Antipsychotic drugs such as quetiapine and haloperidol provide satisfactory results in burn patients [87]. Another aspect associated with the management of burn patients is the alleviation of neuropathic pain; opioids, tramadol, lidocaine, and gabapentin are effective in such patients [88,89,90,91]. If the burn injury remains open, there is a significantly increased risk of the induction of sepsis, and other factors such as the presence of central lines or urinary catheters increase this risk. Therefore, the administration of broad-spectrum antibiotics may reduce this risk, as well as alleviating the side effects of sepsis [1].

## 7. Types of Dressings

So far, dressings ideal for burn wounds have not been developed and implemented on a large scale to allow complete healing without the need for surgical interventions or daily wound care (dressing changes) [4]. Scientists are looking for the gold standard in the burn treatment process, not only to significantly improve and speed up the healing process but also to prevent potential infection. The key to effective treatment is to cleanse the wound as soon as possible after exposure to an external agent. This task is performed with the use of the surgical method (involving cutting out/removing necrotic tissue from the wound) or a conservative method (involving the use of moist specialized dressings such as hydrogels, hydrocolloids, or hydro fibers, mechanical or enzymatic cleansing) [5].

### 7.1. Active Hydrogels for Treatment of Burn Wound Dressings

Hydrogels are a relatively recent group of dressing materials [92]. They can be divided, according to the type of polymer used, into natural and synthetic, and are highly hydrophilic macromolecular networks. Due to their special properties, such as high sensitivity to the physiological environment, the water content in soft tissues, and adequate flexibility, they are an ideal for patients’ convalescence [93]. The use of these dressings has a multipurpose effect, in that they can be applied to virtually all areas of the body, have cooling and wound covering functions, come in many sizes, and can remove heat from the wound through convection and evaporation [94]. In addition, some products are also enriched with agents with anesthetic, nutritional or anti-inflammatory properties. Currently, burn wounds still pose many difficulties. This is because the wound itself requires frequent dressing changes. Some of the dressings adhere tightly to the wound, especially to the burned wound surface. Changing the dressing can lead to new epithelial injuries, delayed healing, and suffering of the patient. In addition, the process of changing the dressing itself is lengthy and, on average, takes one hour.

As is well known, a moist environment is required at the wound site. However, it may also pose a potential risk of infection with microorganisms that have a significant impact on the healing process [95].

### 7.2. Honey-Based Dressings

Honey is a thick, carbohydrate-rich syrup that has been used in traditional medicine since ancient times. It consists mainly of fructose, glucose, and fructooligosaccharides [96]. The composition of honey largely depends on the plants that the insect eats. Flavonoids (including apigenin, pinocembrin, kaempferol, quercetin, galanin, hydrazine, and hesperetin), phenolic acids (including ellagic, coffee, p-coumaric, and ferulic acids), ascorbic acid, tocopherols, catalase (CAT), superoxide dismutase (SOD), reduced glutathione (GSH) and peptides are the main ingredients of natural honey [97]. Currently, honey is widely used in medicine for its antibacterial, antiparasitic, and analgesic properties and its proven effectiveness in respiratory infections [98]. The antimicrobial activity of honey can be ascribed to an acidic pH of 3.2–4.5 depending on the species [99]. The use of honey in the burn treatment process has the advantage of creating a moist environment, preserving the integrity of the burn surface, as it does not adhere directly to it, and providing a bacterial barrier that prevents potential cross-contamination. In a study conducted in mice by Febriyenti et al. a honey film was shown to be very effective in the healing of burns [100]. Modern medicine involves the potential use of natural ingredients in therapy, so it is desirable to use honey in the treatment of burns due to its numerous properties.

### 7.3. Chitin-Based Dressings

Chitin and its derivatives (chitosan) are widespread and inexpensive biological materials isolated from the cell walls of fungi (*Mucoraceae*), insect exoskeletons, and invertebrate skeletons [101]. Chitin was first discovered by Professor Henri Braconnot in 1881 [102]. Chitosan, a product of N-diacylation from chitin, is a biocompatible, biodegradable, non-toxic, antibacterial, non-antigenic, and moisturizing agent [103]. During the process of wound regeneration and healing, chitosan plays an important role in maintaining homeostasis since it can bind with red blood cells, which translates into rapid blood clotting [69]. In addition, it is also responsible for the proliferation of fibroblasts, modeling the functions of inflammatory cells, having a positive effect on the granulation process and cell organization. Used as a semi-permeable biological dressing, it maintains a sterile exudate environment, optimizes healing conditions, and prevents potential scarring and wound contamination. The role of chitin and chitosan as biomaterials has been confirmed in the scientific literature from the last 40 years [102]. Chitin and chitosan stimulate the wound healing process, as confirmed in clinical and veterinary trials. These compounds are used as fibers, powders, granules, sponges, and as composites with cotton or polyester [104]. Treatment with this type of dressings has resulted in a significant reduction in treatment time with minimal scarring in various animals. Examples of chitin-based dressings are Dibucell (producer: Celther Polska) and Beschitin (producer: Unika) [104].

## 8. Vacuum Therapy

Negative pressure wound therapy (NPWT), and VAC (vacuum-assisted closure) therapy, are mechanical methods that use pressure below atmospheric pressure [105]. Low pressure is applied according to the type of wound, its surface, and factors influencing the difficulty of the treatment process, e.g., wound infection [106]. Low pressure is generated by using suction force generated by a pump [105]. During therapy, the cells are mechanically stretched, which stimulates the proliferation process and accelerates the wound healing process [106]. The effectiveness of this method results from its multifaceted operation. Increased local blood flow within the wound, increased collagen synthesis, and mechanisms promoting angiogenesis lead to increased granulation and epithelization, which determine the effectiveness of the therapy [107]. In addition, the influence of other factors, such as a decrease in local swelling or reduction in pathological flora within the wound, have a positive effect on the healing process.

## 9. Use of *Lucilia sericata* Larvae

The coexistence of necrotic tissue in wounds requires cleaning, which is the basis for further treatment [108]. Carefully performed wound debridement promotes wound healing and is associated with an increase in leukocyte phagocytic activity and oxygen tension in the wound, thus ensuring optimal conditions for the regeneration of damaged structures [5]. Additionally, favorable conditions reduce the possibility of potential wound infection, facilitate its evaluation, and enable surgical skin transplantation or the use of optional methods to support the healing process [109]. There may be situations where surgical treatment is indicated but is not possible, e.g., due to the patient’s condition. Then it is recommended to use *Lucilia sericata* larvae therapy (called Larval Debridement Therapy—LDT).

Since ancient times, people have been aware that the larvae of some flies may aid in wound cleansing and disinfection [110]. There is evidence or the use of larvae to heal a wound in images of the Mayan tribes of Central America and the indigenous peoples of Australia. In 1929, a well-known orthopedic surgeon in Baltimore suggested that the use of larvae in the treatment of children with osteitis had positive effects, such as reduction of bacterial count, wound surface alkalization, and reduction of unpleasant odors [111].

**Figure 8:**
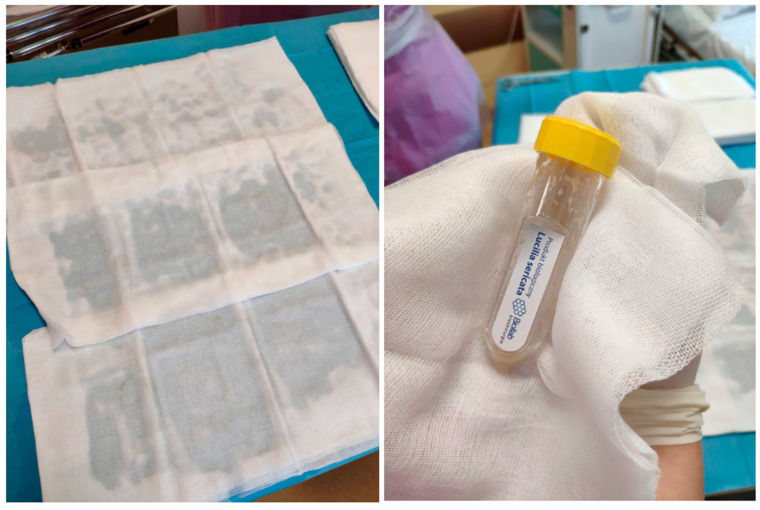
Picture before application of the larvae *Lucilia sericata* to the burn injury.

In 2004, the Food and Drug Administration (FDA) classified *Lucilia sericata* larvae as available, and recommended drugs that can be used for the treatment of chronic wounds [5]. The method cleans cavities from necrotic tissues by controlled therapeutic myiasis [112]. This type of therapy is used in burn patients because they often come to the ward from many different places, which contributes to the appearance of diffuse necrosis. Only *Lucilia sericata* larvae from certified farms can be safely used in medicine [113]. Strict requirements for the use of these larvae are related to the process of their breeding, including complicated egg disinfection procedures with the use of chloramine, povidone, iodine, and sodium hypochlorite to ensure the safety of the medicinal product [114]. When *Lucilia sericata* larvae are introduced into the wound the following can be observed: mechanical removal of necrotic tissue, a bactericidal/bacteriostatic effect, and support of the wound healing process [113]. Scientific reports from the last decade indicate that the physical contact of the larvae with the wound can have a negative effect on the patient in the form of discomfort due to the movement of the larvae in the wound. On the other hand, chemical substances secreted by the larvae initiate the process of eliminating bacteria from the wound and begin the process of remodeling the wound bed. *Lucilia sericata* larvae before application are shown in Figure 8.

## 10. Use of Fish Skin

Early excision and application of Split Skin Grafting is the mainstay of treatment of deep dermal and full-thickness burn injuries to avoid common complications such as sepsis, multi-organ failure, and acute kidney injury [115]. Human cadavers and pig skin are major sources of this temporary coverage. Application of cadaveric and pig skin grafts carries a risk of auto-immune response and risk of viral and bacterial disease transmission. There has recently become available an alternative resource for xenografts using acellular fish skin. Acellular fish skin grafts (FSG) are created by minimally processing fish skin from the Atlantic cod (*Gadus morhua*) [116]. Interestingly, there are no known prion, bacterial, or viral diseases that can be transmitted from North-Atlantic cod to humans; hence, the minimal processing requirements [117]. Acellular fish skin is remarkably like human skin, yet fundamentally different from mammalian-derived matrices, because of the preservation of the structure, lipids, and other soluble components. Mammalian scaffolds require harsh chemical processing to reduce viral and prion transmission risk, but such risks using Atlantic cod (*Gadus morhua*) fish skin are nonexistent [118]. The minimal processing required in the manufacturing of fish skin maintains its three-dimensional structure, as well as its anti-inflammatory and anti-infective properties.

Collagen constitutes an important element of the extracellular matrix that plays a key role in the wound healing process. Keratinocytes and fibroblasts are the main cell types able to deposit collagen in the wound bed. Currently, collagen is mostly extracted from the skin of mammals, such as cattle and pigs [119,120]. Kittiphattanabawon et al. conducted collagen extraction on bamboo shark and blacktip shark with acid-soluble collagen (ASC) and pepsin soluble collagen (PSC) methods [121]. Singh et al. extracted collagen from the skin of Pangasinodon hypothalamus using ASC and PSC methods [122].

In an acute wound model, fish skin has shown faster healing times than porcine intestinal submucosa and dehydrated amnion chorionic membranes [118]. Acellular fish skin CTPs have improved wound healing ability and a low-cost barrier, which are vital characteristics for an effective skin replacement material. Burn care is another area of interest for the application of fish skin. Due to its antibacterial and antiviral properties, as well as its acceleration of 3-D cell ingrowth, fish skin CTPs apply to severe burn victims [123].

## 11. Regenerative Medicine and Burn Wounds

It is known that an endogenous population of somatic (adult) stem cells is involved in a physiological process of cutaneous wound healing. Interestingly, bone marrow-derived mesenchymal stem cells (BMDSCs) were shown to have the ability to migrate to the wound bed and differentiate to skin fibroblasts [124,125]. As many as 20% of the fibroblasts at the site of the healed wound may be BMDSC-derived cells [126]. Adipose tissue-derived mesenchymal stem cells (ADSCs), which are present in a hypodermal layer of the skin, can also differentiate to fibroblasts and participate in the regeneration of the damaged tissue. Furthermore, keratinocytes that are involved in the re-epithelialization of the wound are formed as a consequence of the differentiation of epidermal stem cells located in the interfollicular epidermis and hair follicle bulges [124]. Therefore, it is not surprising that modern regenerative medicine includes stem cell-based therapies for the treatment of burn wounds. The delivery of adult mesenchymal stem cells (MSCs) to the wound bed may help to achieve accelerated healing, and significantly reduced scarring [125,127]. The MSC mechanism of action is associated with paracrine function, and the release of many growth factors (GFs), cytokines, and extracellular vesicles, which are crucial for the promotion of angiogenesis, skin cells’ migration, and proliferation as well as regulation of inflammatory phase during skin healing [128].

According to the available literature, ADSCs are the most frequently used stem cells for the promotion of skin regeneration. Importantly, ADSCs are usually isolated from fat tissue collected by liposuction. After enzymatic digestion of the fat, followed by centrifugation, a stromal vascular fraction (SVF) is obtained, which is subsequently used for the isolation of a pure population of stem cells [129,130]. Apart from ADSCs (10–30%), SVF contains other cells (e.g., pericytes, hematopoietic stem cells, endothelial cells), cytokines, and GFs, having strong immunomodulatory, pro-angiogenic, and pro-healing properties [131,132,133]. Importantly, SVF has been proved to have great potential to be used in the treatment of chronic and burn wounds. Atalay et al. administered SVF via an intradermal route into deep partial-thickness burns in a rat model and demonstrated that SVF could significantly accelerate healing, increase vascularization, and reduce inflammation [130]. Sun et al. [134] revealed that injectable extracellular matrix (ECM)/SVF gel promoted the secretion of pro-angiogenic factors in a murine excisional wound model, significantly enhancing vascularization at the wound bed. Deng et al. successfully used autologous ECM/SVF gel for stem cell-based therapy of chronic wounds of patients in clinics [135]. ECM/SVF gel increased collagen deposition and had strong immunomodulatory as well as pro-angiogenic activity.

In the case of extensive burns (TBSA > 25%) management, pharmacological treatment, and minimally or non-invasive therapies usually fail, or do not bring expected clinical outcomes. Then, regenerative medicine recommends performing excision procedures and skin transplantation. Conventional skin grafting involves the use of autotransplant or allotransplant material harvested from cadavers. In some cases, porcine skin-derived xenografts are also applied [136]. Generally, due to the lowest risk of immune rejection, autografts are the first choice for transplantation. Nevertheless, the application of autografts in patients with very extensive burns is limited, and allogeneic skin transplants are used. Depending on anatomical structure, the skin transplants may be divided into three main types: (1) epidermal skin grafts (ESGs), (2) split-thickness skin grafts (STSGs), and (3) full-thickness skin grafts (FTSGs) [137,138]. For burn wound management, STSGs are the most recommended, and are made of the complete epidermis and part of the dermis that may have different thicknesses (thin STSG—0.2–0.3 mm, medium STSG—0.3–0.45 mm, or thick STSG—0.45–0.75 mm [139]. However, allogeneic skin transplantation is always associated with a high risk of complications, such as disease transmission or graft rejection [140].

## 12. Artificial Skin—Is It a Future Treatment?

Skin tissue engineering, which aims to generate bioengineered artificial skin grafts, may overcome limitations related to the use of autografts or allograft rejection [136,140]. It is an advanced strategy combining technologies typical of engineering of biomaterials and tissue engineering. Artificial skin substitutes act as bioactive wound dressings, whose role is not only to cover the wound but also to facilitate its function and accelerate the healing process. Bioengineered artificial skin should have the ability to supply oxygen to the wound bed, maintain appropriate moisture at the wound microenvironment, accelerate skin regeneration, and protect against infections [136,140,141,142]. Bioengineered skin grafts are produced using various natural or synthetic polymers. Among natural polymers, collagen (type I or III), hyaluronic acid, chitosan, and fibrin are the most frequently used to produce skin grafts. Synthetic polymers include, inter alia, polyethylene glycol (PEG), polyurethane, polylactic-co-glycolic acid (PLGA), polyglactin, and silicone [143,144,145,146,147]. Bioengineered skins may be produced as cellular grafts (biomaterials seeded with the skin cells) or acellular grafts (biomaterial without the cells) [136,140]. Depending on the part of the skin that is mimicked by the artificial grafts, they are classified as epidermal, dermal, or dermo-epidermal [136,148,149].

Epidermal skin substitutes are usually made of the thin biomaterial-based membranes, which may be additionally seeded with keratinocytes in the case of cellular grafts. Thus, biomaterial membranes must be very supportive to cell adhesion and proliferation to allow for effective cell cultivation in vitro. An autologous epidermal graft is generated using the patient’s keratinocytes that are isolated from a skin tissue biopsy and expanded in vitro to form a thin cellular sheet mimicking the epidermal layer [125,150]. The main disadvantage of a cellular epidermal graft is the very long time (2–4 weeks) required for cell cultivation, since keratinocytes are known to have relatively slow proliferation [148,149]. Nevertheless, biopolymer-based membranes (e.g., collagen, fibrin, hyaluronic acid) have proven to overcome this limitation by promoting keratinocyte proliferation under in vitro conditions [148]. Dermal skin substitutes are designed to mimic the dermis of the skin. They are generated either as a biomaterial seeded with the patient’s skin fibroblasts or as a biomaterial-based matrix lacking cells (acellular grafts). After transplantation, an acellular dermal graft performs the function of a scaffold for the endogenous population of fibroblasts, accelerating wound regeneration [148,149]. Dermo-epidermal skin substitutes have been generated to mimic both layers of the skin: dermis and epidermis. Cellular dermo-epidermal grafts often have the form of a bilayered biomaterial seeded with fibroblasts and keratinocytes, or only one type of skin cell (either fibroblasts or keratinocytes). Artificial dermo-epidermal grafts are also produced as 3-D porous cellular layers (with fibroblasts), or acellular dermal layers covered by an acellular thin membrane that plays a role in the epidermis [151,152]. Acellular dermo-epidermal grafts made of only biomaterials are also applied for burn wound management. Nevertheless, considering the crucial role of keratinocyte-fibroblast crosstalk in the wound healing process and re-epithelialization, cellular grafts containing both types of cells appear to be the most desired skin constructs [148,149]. To produce such advanced and functional dermo-epidermal skin substitutes, researchers frequently use techniques such as 3-D bioprinting [153,154] or electrospinning [155]. In recent years, mycelia, the vegetative part of fungi, have generated a growing interest in numerous medical applications due to properties such as high biocompatibility, biodegradability, a self-grown porous structure, and cost-efficiency. Scientific articles have reported that mycelia are considered as suitable scaffolds for fibroblast and keratinocyte growth [156]. Thus, mycelia-derived materials are promising bioscaffolds for artificial skin production in the future.

There are many commercially available artificial skins grafts that may be used for specific clinical use, including treatment of non-healing chronic and burn wounds. Table 1 summarizes commercial skin substitutes designed for the treatment of primarily burn wounds. Although recent progress in the tissue engineering field has led to the development of many promising skin constructs, there are no reports in the available literature on commercial skin grafts that would allow for restoration of sensory and thermoregulatory functions of the healed wound and complete reconstruction of skin appendages (hair, sweat glands) and pigmentation to provide an aesthetic appearance after transplantation. It should also be noted that the generation of bioengineered “living” grafts is a time-consuming and costly process, limiting the clinical application of artificial skin substitutes.

## 13. Summary

Burns are a serious condition, irrespective of the origin, type, depth, or extent of the wound. Burns may occur due to a moment of inattention, and in situations beyond the victim’s control. Because of the clinical conditions they present, they undoubtedly constitute a great challenge for people who provide professional care and help to injured patients.

Patients with burns are at risk of developing various infectious and systemic complications. In addition to local changes, burns can also lead to systemic disturbances in the form of shock and burn disease, which is caused by pain, loss of blood plasma, and poisoning from the absorption of tissue protein breakdown products by the body. The infectious process and the type of infection in a burn is strongly related to the extent and depth of the burn, as well as the general condition of the patient, their age, co-morbidities, and general lifestyle. Our study analyzes information focusing mainly on the clinical aspects of burns and their extent. In addition, the information is closely related to difficulties in the wound healing process, including immunological aspects and pathogens, resulting in treatment and systemic complications. Due to the wide range of treatment methods, burns pose a great challenge to modern medicine associated with new methods of treatment, such as with *Lucilia sericata*, generally available preparations accelerating the wound healing process, and skin substitutes. Pathogen infections remain a huge problem and a challenge for the clinicians. Appropriate treatment results in the disappearance of the disease process; however, when drugs do not bring the desired effect, infection may progress, spread to further tissues, and even throughout the body. Existing infectious complications are difficult to cure in the age of increasing drug resistance.

## Figures and Tables

**Table 1:** Commercially available skin substitutes for the treatment of burn wounds.

Type of the Graft	Commercial Product	Polymeric Composition	Indications for Use	Ref.
Epidermal Cellular: keratinocytes	Laserskin^®^ (Fidia Advanced Biopolymers Ltd., Abano Terme [PD], Italy)	Benzyl esterified hyaluronan derivative	Burn wounds	[157]
Epidermal Cellular: keratinocytes (Keratinocyte sheet prepared based on green method)	JACE^®^ (Japan Tissue Engineering Co., Ltd., Aichi, Japan)	No polymer used	Extensive burn wounds	[158,159,160]
Dermal Acellular	Matriderm^®^ (Medskin Solutiions Dr. Suwelack Skin & Health Care AG, Billerbeck, Germany)	Bovine type I collagen, elastin	Full-thickness burns	[161,162,163,164,165]
Dermal Acellular	Insuregraf^®^ (SK-Bioland Co. Ltd., South Korea)	Porcine type I collagen	Burn wounds	[166]
Dermal Acellular	Integra^®^ (Integra LifeSciences Servoces, USA)	Bovine Type I collagen, chondroitin-6- sulfate	Partial- and full-thickness burns	[162,167]
Dermal Acellular	Nevelia^®^ (Symatese Aesthetics, Lyon, France)	Calf type I collagen	Burn wounds	[168]
Dermal Cellular: fibroblasts	Hyalograft 3D^®^ (Anika Therapeutics, Bedford, MA, USA)	Hyaluronic acid	Deep burns	[169]
Dermal Cellular: human neonatal fibroblasts	Dermagraft^®^ (Organogenesis, Canton, MA, USA)	Polyglactin	Burn wounds	[170]
Dermo-epidermal Acellular	Biobrane^®^ (Smith & Nephew UK Limited, London, UK)	Porcine type I collagen, nylon, silicone	Partial- and full-thickness burns in children	[136,137,138,139,140,141]
Dermo-epidermal Acellular	Hyalomatrix^®^ (Fidia Advanced Biopolymers, FAB, Italy)	Hyaluronic acid, silicone	Burn wounds	[152]
Dermo-epidermal Acellular	PELNAC™ (Gunze Co., Ltd., Kyoto, Japan)	Porcine atelocollagen, silicone	Large acute burns	[171,172]
Dermo-epidermal Cellular: fibroblasts, keratinocytes	Apligraf^®^ (Organogenesis, Canton, MA, USA)	Bovine type I collagen	Partial- and full-thickness burns	[173,174]
Dermo-epidermal Cellular: human neonatal fibroblasts	TransCyte^®^ (Advanced Tissue Sciences, La Jolla, Calif)	Porcine type I collagen, polyglactin	Partial- and full-thickness burns	[151]
